# Empowerment or disempowerment? How generative AI consultation shapes the health decision-making among the new generation of older adults

**DOI:** 10.3389/fpubh.2026.1947697

**Published:** 2026-09-07

**Authors:** Haibei Chen, Zhengyuan Qian, Xianglian Zhao

**Affiliations:** 1 School of Economics and Management, Taizhou University, Taizhou, Jiangsu, China; 2 School of Marxism, Yangzhou University, Yangzhou, China; 3 School of Economics and Management, Nanjing University of Aeronautics and Astronautics, Nanjing, China

**Keywords:** older adults, generative artificial intelligence, health consultation, health decision-making, new generation of older adults

## Abstract

Generative artificial intelligence has lowered the threshold for access to health information, but increased access does not equate to optimal use. While the new generation of older adults benefit from greater convenience, their decision-making risks have not decreased correspondingly, thereby constituting a new health decision-making paradox. To uncover the underlying mechanism by which generative AI health consultations influence decision quality, this study integrates triadic reciprocal determinism and metacognitive theory, constructing an analytical framework with “generative AI health consultation-intelligent information-health decision quality” as the core pathway. Intelligent information is divided into five stages: attention, perception, discernment, trust, and reliance. This study introduces intelligent boundary awareness, algorithmic pandering awareness, and health autonomy orientation as moderating variables, thereby characterizing differentiated effects under varying cognitive and behavioral contexts. Furthermore, this study employs a hybrid methodological approach that combines structural equation modeling and fuzzy-set qualitative comparative analysis to test the model, identifying three types of high-quality health decision-making patterns: trust-reinforced, autonomous adaptive, and cognitive adoption modes. The results indicate that: (1) Generative AI health consultation improves decision quality not merely by increasing the supply of information, but by reshaping new generation of older adults' cognition and engagement patterns with intelligent information; (2) Intelligent boundary awareness, algorithmic pandering awareness, and health autonomy orientation significantly moderate the translation of intelligent information into decision quality, thereby revealing cognitive and agentic boundaries of decision efficacy; (3) The three identified patterns transcend linear explanations based on single factors, health decision quality depends on differentiated alignment among information cognition, technological relationships, and individual autonomy. Therefore, the key to enhancing the efficacy of generative AI health consultations is to shift from tool provision to empowerment, with a primary focus on strengthening intelligent information discernment, rational trust, and autonomous decision-making capabilities in the new generation of older adults.

## 1 Introduction

### 1.1 Research background

Generative AI is reshaping how the new generation of older adults access and utilize health information. As a cohort that integrated into the digital society comparatively early, they have already begun to proactively leverage generative AI to seek daily health advice on topics such as dietary planning, sleep improvement, home exercise, and emotional regulation. Unlike traditional search engines, generative AI integrates user needs through natural language interaction and provides highly personalized action plans directly (1), offering new pathways for the new generation of older adults to manage their daily health and enhance quality of life. Against the dual backdrop of intensified population aging and rapid digital technology development, the real-world significance of this phenomenon is becoming increasingly apparent. The population of the new generation of older adults, who tend to have higher levels of education, richer digital experience, and stronger willingness for social participation, continues to grow and is becoming an important segment of the older adults demographic (2). Meanwhile, generative AI has rapidly shifted from a specialized technical tool to an immediately usable information service, permeating health information access and health management on a large scale, making the new generation of older adults an emerging key focus in digital health research.

However, while generative AI expands access to health information, it has not simultaneously resolved issues of information authenticity and decision-making reliability. Compared to traditional online misinformation, AI-generated misleading content is often linguistically fluent, structurally complete, and logically coherent, which makes it easy for individuals to mistake fluency for professionalism, equate completeness of explanation with authenticity, and perceive superficial personalization as advice tailored to their own circumstances (3). For the new generation of older adults, generative AI health consultation can broaden their channels for obtaining health information, enhance their sense of self-efficacy and health autonomy, and thereby promote more rational and self-serving health decisions; however, such consultations may also interfere with their judgment due to factors like misinformation and cognitive load, leading to biased decision-making (4). Evidently, the ability of the new generation of older adults to actively use generative AI does not necessarily equate to the ability to judge information accurately or use it scientifically. Although stronger device operation skills, information search abilities, and confidence in technology use can assist them in daily health management, these traits may also increase their frequency of use and exposure to erroneous information. In the absence of effective judgment regarding the authenticity, applicability, and potential risks of the information, incorrect or misleading health advice may be adopted and transformed into inappropriate health decisions, negatively impacting daily health management. Therefore, enhancing the ability of the new generation of older adults to assess and appropriately use health information generated by artificial intelligence has become an urgent issue to be addressed in the development of digital health.

Compared to the traditional older adults, the new generation of older adults possesses a stronger educational background, greater cognitive reserves, and better adaptability to digital technologies, enabling them to understand and use generative AI more readily and integrate it into their daily health information acquisition and health management. However, stronger cognitive and technological abilities do not necessarily mean they can always accurately assess the authenticity and applicability of AI-generated information, and there are significant individual differences in information judgment abilities (5). Especially after continuous use and the formation of dependence, it becomes difficult to promptly adjust their level of trust based on the accuracy of information. Improved operational skills have increased the participation of the new generation of older adults in generative AI health consultations and their exposure to misleading information, while insufficient critical evaluation abilities make it difficult for them to accurately calibrate their trust in AI, thus leading to inappropriate health decisions. Evidently, the key issue faced by the new generation of older adults is not a general lack of information judgment ability but the potential misalignment between technological proficiency, cognitive advantages, and the ability to assess generative AI health information. Existing studies have mainly examined the individuals' acceptance and use of generative AI, as well as the impact of digital health literacy and trust in AI, providing a foundation for understanding intelligent health consultation behaviors among older adults (6). However, these studies have not adequately focused on this specific group of proactive generative AI users among the new generation of older adults, nor have they systematically revealed how AI misinformation is gradually transformed into inappropriate adoption through information discernment, trust and dependence. Bridging this gap is key to explaining why the new generation of older adults may use generative AI but may not be able to make scientifically sound judgments, and to further improve the health decision quality.

Based on the aforementioned practical challenges and research gaps, this study focuses on the following questions: In the process where the new generation of older adults proactively use generative AI to obtain daily health advice, why does stronger digital literacy facilitate their use of generative AI, yet not necessarily improve their ability to identify erroneous health information? When AI recommendations are misleading, which factors cause the new generation of older adults to become overly reliant on or mistakenly adopt them? Among the new generation of older adults who actively participate in digital intelligent contexts and consult generative AI for health information, how do their attention, perception, discernment, trust, and reliance on intelligent information gradually evolve and ultimately influence the quality of their health decision-making?

### 1.2 Motivation and contribution

To gain deeper insights into the underlying mechanisms of health decision quality among the new generation of older adults, this study integrates triadic reciprocal determinism and metacognitive theory to propose the core pathway of “generative AI health consultation-intelligent information-health decision quality” elucidating how health consultations exert profound influences on health decisions through intelligent information. Furthermore, intelligent information is subdivided into five progressive stages: attention, perception, discernment, trust, and reliance systematically depicting the psychological evolution of the new generation of older adults as they move from initial information exposure to deepening dependence on human-AI interaction. Simultaneously, three moderating variables are introduced: intelligent boundary awareness, algorithmic pandering awareness, and health autonomy orientation, to reveal differentiated pathways through which intelligent information affects health decision quality under varying cognitive and behavioral contexts and to identify key conditions under which misleading recommendations trigger overdependence and erroneous adoption. Accordingly, a theoretical model is constructed in which generative AI health consultation, via multistage internalization of intelligent information, impacts the health decision quality of the new generation of older adults. Subsequently, a hybrid methodology combining structural equation modeling and fuzzy-set qualitative comparative analysis is employed to conduct an overall validation of the model and to delve into the diverse configuration pathways leading to high- and low-quality health decisions under different combinations of conditions. This study strives to clarify the capability paradox and the risks of intelligent dependence, while helping the new generation of older adults enhance their ability to discern and reflect on intelligent health information, develop rational trust, maintain moderate dependence, and preserve decision-making autonomy in generative AI health consultations, thereby improving health decision quality and well-being in the era of digital intelligence.

Centered on the aforementioned research questions and analytical framework, this study's main contributions are reflected in the following three aspects. (1) From a process-oriented perspective, it uncovers the dynamic evolution by which the new generation of older adults in generative AI health consultations progress from initial information exposure to deepening reliance. This deepens the understanding of the paradox whereby digital literacy promotes use without necessarily enhancing information discernment and expands theoretical explanatory pathways for the decision-making behaviors of the new generation of older adults in the context of generative AI health consultations. (2) The analytical focus shifts from traditional older adults groups to the new generation of older adults who possess stronger digital literacy and actively integrate intelligent technologies, breaking through the perspective that simplistically regards the older adults as passive technology adopters. By focusing on information assessment, trust-building, and dependence formation during the active use of generative AI for health advice, the study reveals the unique mechanism of being able to use but not necessarily discern, thereby enhancing the theoretical explanatory power of research on AI health behaviors in the new generation of older adults. (3) It identifies multiple configurational pathways for the formation of health decision quality under different combinations of cognitive and behavioral conditions, providing a more flexible analytical tool for understanding the complexity of intelligent health decision-making among the new generation of older adults. On this basis, it identifies key risk points, such as misjudgment, blind faith, and excessive dependence, thereby offering a foundation to help the new generation of older adults develop rational trust, moderate reliance, and maintain decision-making autonomy in intelligent health consultations, ultimately improving their health decision quality and well-being in the digital intelligence era.

## 2 Literature review

### 2.1 Health information behavior of the new generation of older adults

As a transitional group between digital natives and digital immigrants, the digital health literacy and technology adoption behaviors of the new generation of older adults are key to understanding their health information behaviors. Compared with traditional older adults, they demonstrate a more proactive attitude toward technology, viewing it not only as an important aid for healthy aging but also as actively employing it for self-health management. However, despite more frequent exposure to technology, this group's digital health literacy remains lower than that of younger groups, which may limit their ability to effectively utilize digital health tools. Further research indicates that the older adult population exhibits significant heterogeneity in terms of age, education, prior technological experience, and attitudes toward technology (7, 8). Therefore, unlike the traditionally digitally disadvantaged older adults, the new generation of older adults already possesses relatively rich experience with the Internet and smart devices, and their health information challenges are shifting from whether they can access information to how they can autonomously obtain, compare, evaluate, and use information. However, proactive information acquisition does not necessarily lead to better outcomes. Studies have found that autonomous online health information seeking may exacerbate health anxiety through perceived information quality, while proxy seeking serves to alleviate it (9). This suggests that once the new generation of older adults possesses autonomous acquisition abilities, research must move beyond dimensions such as access, frequency of use, or literacy levels and instead examine how they select information channels, comprehend content, assess reliability, and integrate information into subsequent health actions.

Existing research has primarily focused on channels such as website search, social media, and mobile health applications (10, 11), with limited attention given to the emerging information behavior in which the new generation of older adults proactively pose health-related questions to general-purpose generative AI and obtain personalized responses through ongoing dialogue. In traditional online search scenarios, users must filter among multiple existing information sources; however, in the context of generative AI, the system directly generates and organizes responses. This shift makes it an urgent research issue to understand how the new generation of older adults comprehend and evaluate AI-generated content and provides a crucial entry point for extending research on older adults' health information behavior to the study of generative AI health consultations.

### 2.2 Influencing factors of generative AI consultation

Generative AI has accelerated the shift among the new generation of older adults from traditional online search to proactive information acquisition, a transition driven jointly by technological features, information quality, and individual characteristics. On a technological level, the natural language interaction offered by generative AI provides a human-like experience that appeals to new generation of older adults accustomed to interpersonal communication (12); however, interface usability, operational convenience, and response speed are equally crucial. While technological features shape initial perceptions, information quality is fundamental to building trust. The accuracy, completeness, timeliness, objectivity, and comprehensibility of information directly determine its value for health decision-making (13). Nevertheless, older users lacking specialized knowledge may find it difficult to discern false information, potentially leading to errors in their decision-making. It is noteworthy that the intuitiveness of information often plays a greater role than the source itself: advice consistent with existing beliefs is more readily accepted, while even accurate information may be questioned if it conflicts with expectations (14). Therefore, balancing scientific rigor with intuitive comprehensibility is essential for fostering trust.

The impact of technology and information is not independent but rather gives rise to differentiated effects through individual characteristics. AI usage experience, health literacy, and risk perception profoundly influenced trust and adoption. Studies show that perceived susceptibility, trust in generative AI, AI literacy, and health information literacy positively affect older adults' willingness to adopt generative AI as a replacement for search, while perceived privacy risk negatively affects this trust. The perceived service experience can indirectly promote this willingness by enhancing trust. From the perspective of deep cognitive mechanisms, AI literacy and health information literacy form a dual driving force for adoption; individual differences not only directly impact willingness to adopt, but also exert indirect effects by altering evaluations of technology and information (15).

In summary, the adoption of generative AI health consultation by the new generation of older adults is a multi-layered process triggered by technology, validated through information quality, and moderated by individual characteristics. The interaction among these factors provides a foundation for further exploration of how they evaluate generative AI health responses.

### 2.3 The impact of generative AI consultation on health decision-making

The direct impact of generative AI consultation on health decision-making among the new generation of older adults remains to be thoroughly investigated; however, existing research has identified potential pathways. On the positive side, compared to traditional search engines, generative AI is less time-consuming and offers better perceived effectiveness, efficiency, and overall user experience (16). This means that generative AI quickly integrates and distills information, improving the efficiency of information acquisition and laying an informational foundation for reliable decision-making. Furthermore, convenient, personalized, and low-threshold access to health information helps enhance the health literacy and self-efficacy of the new generation of older adults (17). When these individuals can access and understand complex medical information at a relatively low cognitive cost, their sense of control over their health increases, motivating them to participate more actively in health management. At the same time, when the health advice generated by generative AI is highly accurate, comprehensive, and aligned with the specific health status of the older adults, it is more likely to gain their trust and be adopted, thereby improving adherence to healthy behaviors (18). However, generative AI may also produce information that appears reasonable but is misleading, a potential risk that warrants attention. For older adults users lacking professional medical judgment, making decisions based on distorted information could jeopardize their health. Therefore, ensuring the accuracy and reliability of information is a fundamental prerequisite for guaranteeing the quality of health-related decisions. In addition, algorithmic opacity may affect decision-making autonomy. Thus, generative AI health consultation is not simply a tool for assistance but rather involves multiple relationships, including information efficiency and decision quality, cognitive support and algorithmic dependence, and behavioral adherence and decision autonomy.

Overall, generative AI consultation has both facilitating and inhibiting effects on health decision-making among the new generation of older adults; its ultimate impact depends not only on the quality and transparency of information but also on the discrimination and critical use by the new generation of older adults.

### 2.4 Research gap

Although existing research has explored the use of generative AI for health consultation among the older adults from perspectives such as technology acceptance, model accuracy, and digital health literacy, it lacks explanatory frameworks that integrate AI information characteristics, user judgment processes, and health decision outcomes. Firstly, current literature mainly focuses on digitally disadvantaged older adults, addressing issues such as the digital divide, technology anxiety, and barriers to use (19), while paying insufficient attention to the new generation of older adults who proactively adopt generative AI as a health consultation tool, particularly failing to reveal the latent risk of overreliance underlying their proactive use and high trust. Secondly, most research on the risks of AI health information centers on dedicated health consultation systems or health management applications (20); however, generative AI is not specifically designed for health decision-making scenarios, yet can produce seemingly plausible but not necessarily accurate health advice. There is a lack of systematic studies on how the new generation of older adults assesses the credibility and usability of such information. In addition, most studies are concentrated on usage intention or adoption intention (21), with limited and insufficient explanations regarding post-adoption processes such as information reliability identification, trust calibration, and the translation from information acceptance to actual health decision-making. Meanwhile, current intervention approaches are mainly oriented toward enhancing digital health literacy and promoting AI-enabled access to health information, overlooking the more urgent practical need to help scientifically identify AI-generated health information and prevent misleading information from infiltrating actual health decision-making.

In response to the aforementioned shortcomings, the innovations of this study are reflected in the following aspects. First, the research focus shifts from traditionally digitally disadvantaged older adults to the new generation of seniors who proactively use generative AI for health consultations, with particular attention to their potential for overreliance stemming from high initiative and trust. This addresses the insufficient attention paid to this group in the existing research. Second, the research context transitions from dedicated health consultation systems and health apps to generative AI, focusing on the unique risks associated with generating health-related responses in non-health-specific scenarios, thereby filling the gap in the types of tools examined in the current research. Third, the investigation moves the research mechanism forward from usage intention and adoption intention to cover the full chain of reliability identification, trust calibration, and health decision-making, revealing how surface features of information, such as fluency and personalization, influence older adults' reliability judgments and decision-making pathways, and deepens our understanding of cognitive and behavioral mechanisms after adoption. Fourth, the research objective shifts from AI empowerment in health information acquisition to the scientific identification of AI-generated health answers, aiming to construct critical health literacy intervention pathways for conversational AI environments and provide effective empirical support for helping older adults resist misleading information.

## 3 Research framework and hypothesis

### 3.1 Theory framework

Generative AI health consultation not only provides new avenues for the new generation of older adults to access health information and make autonomous decisions, but also drives a shift in health decision-making from traditional interpersonal consultation and one-way information search toward continuous, interactive, and personalized human-AI collaboration. However, strong digital literacy and a high level of technological engagement do not necessarily enable individuals to effectively identify false health information; instead, ongoing interactions may lead to trust drift and deepening dependence, thereby creating a paradox in health consultation in which intelligent empowerment and decision-making risks coexist. This study aims to reveal that this paradox is rooted in the ongoing coupling of individual cognitive abilities, intelligent information characteristics, and the human-AI interaction processes. With a solid foundation in digital engagement, high information-seeking proactivity, and a heightened sense of health autonomy, the new generation of older adults is gradually shifting toward becoming intelligent health decision-makers who can proactively raise questions, evaluate recommendations, and take action accordingly. In this study, the new generation of older adults refers to a relatively younger segment of the older population with greater exposure to digital technologies, a basic foundation of digital literacy, and stronger acceptance of emerging technologies. They are generally more willing and able to engage autonomously in digital life and to incorporate emerging technologies, including generative AI, into health information seeking and health decision making. The process by which they use generative AI for health consultation is, in essence, a dynamic cognitive processing cycle in which intelligent information enters the cognitive system and is gradually transformed into the basis for decision-making. On this basis, this study moves beyond the traditional paradigm of interpreting AI health behavior solely through technology adoption or willingness to use, and instead takes “generative AI health consultation-intelligent information-health decision quality” as the central analytic thread, constructing an integrative framework linking technology use, cognitive processing, and decision outcomes. The establishment of this framework requires examining both how external human-AI interactions shape the internalization of information and how internal cognitive regulation further influences decision outcomes, thereby introducing triadic reciprocal determinism and metacognition theory to jointly elucidate the mechanisms of action on both external interaction and internal cognition levels.

The triadic reciprocal determinism theory emphasizes the continuous interaction and mutual shaping of individuals, behaviors, and environments (22), providing a holistic perspective for understanding the dynamic process of generative AI health counseling. Metacognitive theory, on the other hand, focuses on individuals' awareness, evaluation, and regulation of cognitive activities, which helps reveal how intelligent information transforms from external input into internal judgment and decision-making basis (23). In this study, the cognitive characteristics and digital literacy of the new generation of older adults constitute individual factors, proactive health consultation forms the behavioral factor, and the content, feedback, and interaction modalities provided by generative AI comprise a new intelligent information environment. The ongoing interaction among these three drives the health consultation process through the cognitive processing stages of attention, perception, discernment, trust, and reliance. Specifically, attention to AI-generated health information marks its entry into the cognitive domain; perception of AI-generated health information reflects an individual's initial understanding of its value and usability; discernment of AI-generated health information involves scrutiny of the information's authenticity and appropriateness; trust in AI-generated health information pertains to the judgment of the reliability of intelligent recommendations; and reliance on AI-generated health information signifies the transformation of intelligent information from a reference source to an actual basis for decision-making. Thus, the impact of generative AI health counseling on decision quality can be seen as a continuously evolving process in which human-AI interaction deepens cognitive processing and ultimately influences decision behavior. However, this process does not necessarily lead to rational decisions; its outcomes depend on whether individuals can recognize technological limitations, reflect on algorithmic feedback, and maintain autonomy in their decision-making. Based on the regulatory function of metacognition theory, this study introduces three moderating variables: intelligent boundary awareness, algorithmic pandering awareness, and health autonomy orientation. Intelligent boundary awareness refers to an individual's awareness of the limits, uncertainties, and potential errors of generative AI. Algorithmic pandering awareness refers to an individual's recognition of the tendency of generative AI to align feedback with personal positions, preferences, and expectations. Health autonomy orientation is the tendency to insist on independent judgment and assume responsibility for health decision-making. These three aspects correspond to technological boundary awareness, reflective algorithmic interaction, and maintenance of decision-making autonomy, constituting key boundary conditions for the internalization of intelligent information, which can explain why similar health consultation experiences may result in varying degrees of trust, dependence, and decision outcomes.

**Figure 1:**
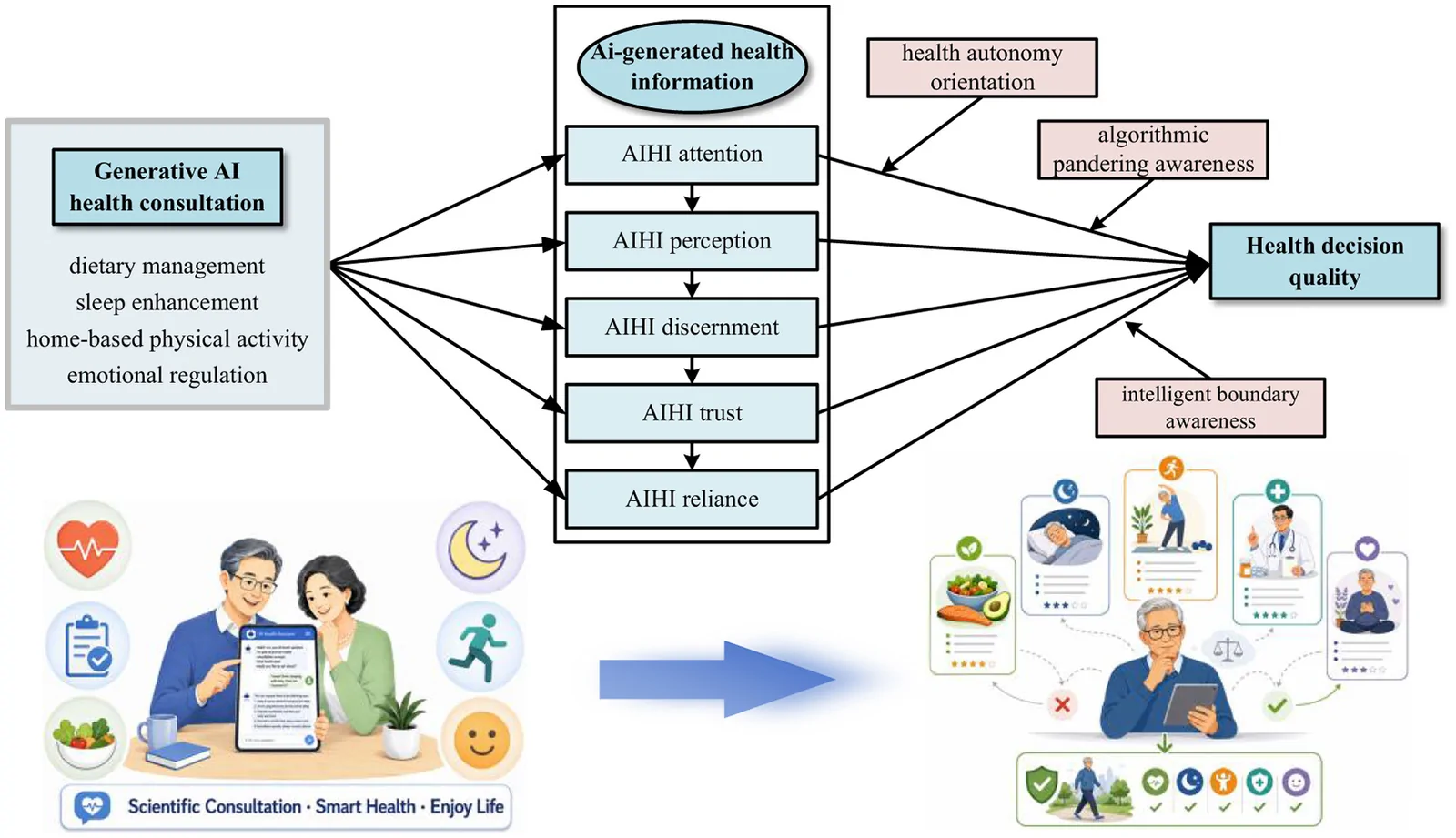
Theoretical framework of health decision-making among new generation older adults.

In summary, this study integrates triadic reciprocal determinism and metacognition theory to develop an integrative analytical framework centered on “generative AI health consultation-intelligent information-health decision quality” as the primary axis. The framework takes the five-stage internalization process of intelligent information as intrinsic mechanisms, and intelligent boundary awareness, algorithmic pander awareness, and health autonomy orientation as boundary conditions. This framework unifies human-AI interaction, cognitive processing, and decision outcomes within a single analytical process, aiming to reveal that while generative AI empowers health decision-making among the new generation of older adults, it may also, owing to insufficient metacognition, lead to misplaced trust, excessive dependence, and reduced decision quality. Based on the above theoretical integration, this study constructs a theoretical framework for how generative AI health consultation influences the quality of health decision-making among the new generation of older adults, as shown in Figure 1.

### 3.2 Research hypothesis

#### 3.2.1 Main path and mediating effect

This study contends that generative AI health consultations do not directly determine the quality of health decision-making among the new generation of older adults; rather, their impact is gradually realized through the internalization of intelligent information by the user. Health consultation constitutes the starting point of this internalization process, while intelligent information serves as the key intermediary transmitting the effects of human-AI interaction to decision outcomes. The quality and evolutionary stage of this internalization ultimately determine whether the technology functions as decision support or, owing to misplaced trust and overreliance, negatively affects decision quality. Accordingly, the “generative AI health consultation-intelligent information-health decision quality” pathway forms the core mechanism of this study. Intelligent information can be further subdivided into five dimensions: attention, perception, discernment, trust, and reliance.

(1) **generative AI consultation** **→** **attention to AI-generated health information** **→** **health decision quality**

Attention to AI-generated health information is a prerequisite for information to enter the health decision-making process. Compared with traditional health searches, generative AI transforms scattered information-seeking into focused interactions centered on specific health issues through instant responses, continuous dialogue, and personalized presentation, making sustained attentional engagement easier to achieve (24). For the new generation of older adults, who generally have a good digital foundation and a strong awareness of proactive information acquisition, actively seeking consultation further reinforces selective attention to relevant symptoms, risk alerts, and recommended actions, allowing limited cognitive resources to be concentrated on information closely related to the current issue (25). Health consultations strengthen the salience and concentration of information, reducing key information omissions, laying a cognitive foundation for subsequent understanding, judgment, and choice, and ultimately influencing decision quality.

(2) **generative AI consultation** **→** **perception of AI-generated health information** **→** **health decision quality**

The perception of AI-generated health information is the process of meaning construction, in which information presented externally is transformed into the basis for internal decision-making. The core value of generative AI lies not only in providing information, but more importantly in reshaping the way users understand health information through natural language interaction, contextualized explanations, and real-time follow-up questions (26). Although the new generation of older adults possesses a certain degree of digital experience, they may still encounter barriers to knowledge transfer when faced with technical terminology, complex risk probabilities, and weighing multiple options. Generative AI can reorganize information based on specific issues, transforming specialized content into expressions that are more understandable, relevant, and actionable, thereby influencing users' subjective perceptions of information value and applicability (27). A clear and accurate perception facilitates a fuller problem representation, enabling individuals to effectively link external information with their health conditions, prior experiences, and real-world constraints, thus improving the alignment between decision-making criteria and actual needs.

(3) **generative AI consultation** **→** **discernment of AI-generated health information** **→** **health decision quality**

Discernment of AI-generated health information is the key cognitive filtering step linking health consultation to high-quality decision-making. Generative AI offers both information richness and content uncertainty; its fluent language and complete responses may obscure factual errors, insufficient evidence, and contextual mismatches (28). Therefore, the effectiveness of health consultation hinges not on the quantity of information acquired but on the ability to identify which information is worth adopting. The stronger digital skills of the new generation of older adults enable them to pursue follow-up questions, compare different explanations, and seek supporting evidence; however, only by further transforming this operational capability into careful scrutiny of information sources, logical consistency, adequacy of evidence, and individual applicability can effective discernment be achieved (29). In essence, the process of discernment fulfills a cognitive filtering function before information enters decision-making, reducing the potential for superficially credible information to mislead judgments and intercept erroneous information before it directly influences decisions.

(4) **generative AI consultation** **→** **trust in AI-generated health information** **→** **health decision quality**

Trust in AI-generated health information is the key step that transforms the understanding of information into its adoption. Health information can only truly enter an individual's evaluative framework when it is attributed a certain degree of credibility. Generative AI, through continuous responses, linguistic consistency, personalized feedback, and human-like interactions, gradually builds a sense of trust over repeated consultations, making trust no longer derive solely from technical authority (30). For the new generation of older adults, extensive digital engagement experience means they do not accept information simply because it is delivered by AI, but are more likely to form trust judgments based on the professionalism, consistency, and alignment with their own experience found in the answers (31). Calibrated trust that matches information quality can reduce uncertainty and cognitive costs in health decision-making, allowing valuable intelligent advice to receive appropriate decision weights. Conversely, misplaced trust may amplify the impact of incorrect information. The degree to which trust is calibrated directly affects the quality of health-related decisions.

(5) **generative AI consultation** **→** **reliance on AI-generated health information** **→** **health decision quality**

Reliance on AI-generated health information reflects the extent to which AI is integrated from cognitive reference into actual decision-making. It is the transmission mechanism that most directly links decision quality to behavioral outcomes. The low cost, immediacy, continuous availability, and high interactivity of generative AI are steadily lowering the threshold for repeated consultations, leading the new generation of older adults to gradually incorporate it into their routine health judgments and choices of action (32). Especially when faced with health issues characterized by complex information, high professional barriers, or significant decision uncertainty, ongoing consultations may see generative AI evolve from a supplementary information source into a stable decision support tool (33). Unlike trust, which is mainly manifested as an assessment of credibility, reliance directly determines the weight of intelligent advice in the final decision. Appropriate reliance enhances the efficiency of information utilization and decision consistency. However, if it surpasses individual discrimination abilities and the actual capacity boundaries of AI, it may lead to judgment errors and the adoption of incorrect recommendations. The appropriate degree of reliance determines whether intelligent empowerment can be transformed into a decision-making advantage.

#### 3.2.2 Moderating effect

(1) **The moderating effect of intelligent boundary awareness on intelligent information and health decision quality**

Health advice generated by generative AI is often coherent, complete, and highly definitive; however, conveying definitiveness is not equivalent to content accuracy. Although the stronger digital experience of the new generation of older adults can enhance their proficiency in using such systems, it may also reinforce the overestimation of intelligent system capabilities. Intelligent boundary awareness helps them distinguish between technologies that provide references and those that can substitute for professional judgment, thereby recalibrating the weight of intelligent information in decision-making (34, 35). With higher boundary cognition, the new generation of older adults is more likely to consider AI-generated outputs as sources of information that require verification through professional opinions, personal health history, and real-world context, making them more likely to translate into effective decision support. Conversely, insufficient boundary cognition makes it easy to misinterpret fluency and immediacy as professionalism and reliability, amplifying the risk of erroneous information influencing decision-making. Accordingly, intelligent boundary awareness strengthens the positive impact of high-quality intelligent information and mitigates the adverse effects of information distortion.

(2) **The moderating effect of algorithmic pandering awareness on intelligent information and health decision quality**

Generative AI adjusts its responses based on the way questions are posed, pre-existing stances, and tendencies of expression. While this adaptive interaction can enhance the communication experience, it may also become a technological amplification mechanism of confirmation bias. The new generation of older adults demonstrates strong information proactivity and high autonomy in expression, tending to obtain answers aligned with their needs through iterative questioning; as a result, they are more likely to experience ongoing affirmation of their viewpoints in repeated interactions. Algorithmic pandering awareness prompts them to recognize that being affirmed does not equate to being substantiated, thus maintaining necessary distance from information that closely matches their expectations and actively conducting cross-verification (36). Heightened algorithmic awareness weakens erroneous associations between emotional identification, position alignment, and information authenticity, making intelligent information's influence on health decision-making more dependent on evidence quality than interaction comfort.

(3) **The moderating effect of health autonomy orientation on intelligent information and health decision quality**

The low barrier to entry and high accessibility of generative AI have enabled its integration into everyday health assessments. However, the ultimate weight of such information in decision-making depends on whether decision-making autonomy is retained or not. Members of the new generation of older adults generally demonstrate strong engagement in health management, and those with higher levels of health autonomy are more likely to treat AI-generated advice as an auxiliary resource, integrating it with personal experience, professional opinions, and actual preferences to make comprehensive judgments, thus avoiding the transfer of decision-making responsibility to technological systems. In contrast, individuals with lower health autonomy orientation, even if they possess some capacity to discern information, may still over-rely on AI-generated recommendations in pursuit of convenience, a sense of certainty, or to shift responsibility (37). Therefore, health autonomy orientation regulates the translation of AI-generated information into decisions, making it more likely to serve as a judgment-supporting tool rather than supplanting the authority of individual decision-making.

## 4 Research design

### 4.1 Empirical methodology

Generative AI health consultations can enhance access to health information and decision support capabilities but may also compromise decision quality due to misinformation, misjudgment, and overreliance. Accordingly, this study does not focus on the simple effect of a single variable but rather on the complex interaction system encompassing human-AI interaction, information internalization, cognitive modulation, and the interplay of multiple conditions. Structural equation modeling and fuzzy-set qualitative comparative analysis were employed to systematically test the core hypotheses. SEM can simultaneously handle latent variable measurement and structural path estimation, test direct, mediating, and moderating effects, and characterize the direction and strength of each path, thereby revealing the net effect of intelligent information internalization and its boundary conditions (38). Simultaneously, considering that the health decisions of the new generation of older adults are shaped jointly by cognitive characteristics, information internalization status, and usage scenarios, relying solely on average linear effects is insufficient to explain the multiple pathways leading to the same outcome. Therefore, fsQCA is further introduced to examine the synergistic effects of multiple conditions and causal asymmetry from a configurational perspective (39), identifying different configurations of conditions for high-quality and low-quality health decisions. The two methods address which variables have a significant impact and which combinations of conditions jointly lead to the outcome, thus offering a complementary analysis of health decision-making.

### 4.2 Questionnaire design

The questionnaire design primarily drew on established measurement instruments and was adapted to the specific context of the new generation of older adults using generative AI to obtain health advice. To prevent respondents from answering based on general perceptions of AI, the questionnaire provided necessary explanations of generative AI health consultation before the formal measurement, requiring respondents to make judgments based on their actual usage experience. Screening questions were also set to ensure that the participants met the research subject criteria and had genuine consultation experience. All items are consistently anchored to the processes of proactive consultation, intelligent information internalization, and subsequent health decision-making, and are contextually adjusted according to features such as generative AI's continuous dialogue, personalized feedback, content generation, and information uncertainty to ensure alignment between measurement content and research constructs.

In terms of procedure, the questionnaire design sequentially underwent scale selection, contextual revision, expert review, and pilot testing. First, items with good reliability and validity, and appropriate for the research context, were selected for each variable based on their theoretical implications. The expressions and application scenarios were adjusted as needed without altering the original construct's meaning. Next, experts in relevant fields were invited to review the questionnaire from the perspectives of conceptual alignment, content completeness, and expression accuracy. Items with strong technical terminology, semantic ambiguity, or high potential for misunderstanding, especially considering the cognitive and linguistic characteristics of the new generation of older adults, were revised accordingly. During pilot testing, particular attention was paid to respondents' understanding of the research context, item meaning, and response logic, and the questionnaire wording and structure were optimized based on feedback. The final questionnaire was filled out anonymously, emphasizing that there were no right or wrong answers to the questions. By arranging the order of items rationally and appropriately separating measurements of different variables, the common method bias was reduced. The final questionnaire, established through the above procedures, ensured that the measurement tool demonstrated good content validity and had contextual applicability. The main items are listed in Table 1.

**Table 1:** Questionnaire items.

Number	Item content
HC1	In daily life, I often use generative AI to answer health-related questions.
HC2	Whether for daily health maintenance or exercise recommendations, I habitually consult generative AI.
HC3	Compared with consulting books, I prefer to quickly obtain health plans through generative AI.
IA1	When reading the responses from generative AI, I intentionally look for key points related to my health status.
IA2	I will pay particular attention to specific food combinations, taboos, or concrete health-related values mentioned by generative AI.
IA3	Even if generative AI provides a very long answer, I will still read through the most important suggestions patiently.
IP1	I think the tone in which generative AI communicates is very professional and sounds highly reliable.
IP2	The logic behind the recommendations provided by generative AI is clear, making them highly convincing.
IP3	The text provided by generative AI is clear and easy to understand, making it effortless to read.
ID1	I would be wary of whether the advice given by generative AI is intended to promote a certain health supplement.
ID2	When the recommendations provided by generative AI sound dubious, I consider whether they are scientifically valid or not.
ID3	I will probe the reliability of generative AI by asking.
IT1	I believe that the big data analytics behind generative AI are more scientific than individual experiences.
IT2	I believe that the health information provided by generative AI is generally objective and neutral.
IT3	When facing uncertain health issues, I believe that generative AI is a reliable assistant.
IR1	If the recommendations of generative AI differ from my own experience, I will choose to follow them.
IR2	Since generative AI has made things quite clear, I do not think it is necessary to verify with multiple sources.
IR3	I am accustomed to following health advice generated by generative AI directly.
BA1	I am well aware that generative AI is merely a program and may make mistakes when dealing with complex health issues.
BA2	I understand that just because generative AI sounds eloquent does not mean that everything it says is true.
BA3	I can sense that generative AI is actually “beating around the bush” when answering certain difficult questions.
PA1	I feel that generative AI sometimes says what I want to hear, echoing my thoughts.
PA2	I worry that generative AI might hide health risks I do not want to hear about to keep me happy.
PA3	I understand that the way generative AI makes recommendations may be because they think I like to watch.
HA1	When it comes to major health matters, I insist on making my own decisions; generative AI are for reference only.
HA2	I dislike being completely led by the nose by generative AI for health management.
HA3	I believe that my body is my own to control and I should be the one making decisions about it.
HD1	After making adjustments based on the recommendations of generative AI, I feel that my physical condition has improved.
HD2	I feel confident about the health choices I make after consulting the recommendations of generative AI.
HD3	With the help of generative AI, I can more effectively avoid the pitfalls of pseudo-health practices.

### 4.3 Sample selection and data collection

This study targeted the new generation of older adults, specifically the relatively younger group who possess basic digital literacy, are able to independently use generative AI for health consultations, and incorporate relevant information into their daily health management. Operationally, eligible participants were required to meet the age criterion specified for the new generation of older adults in this study and to have prior independent experience using generative AI for health consultation. Basic digital literacy was reflected in their ability to independently engage with generative AI for health-related information seeking, rather than being treated as a separate screening threshold. Given the specific requirement that participants have prior experience with generative AI health consultations, a sampling strategy combining convenience and referral-based recruitment was adopted. The questionnaire was distributed and collected using a combination of online and offline methods. Online distribution was conducted by disseminating links via social media, interest communities, and existing social networks, with the help of respondents forwarding the survey to eligible new generation of older adults. Offline recruitment and completion took place in venues where older adults gather, such as community centers, senior universities and activity centers. To improve the alignment between the sample and the research topic, screening questions were set before the formal questionnaire items to verify respondents' age and experience with generative AI health consultations, as described above. Only those meeting both eligibility criteria were able to proceed to the formal questionnaire. Online questionnaires were independently completed by the respondents following the unified instructions. In the offline survey process, researchers provided necessary equipment and operational assistance according to respondents' actual needs, such as helping access the questionnaire, navigate pages, and submit responses, but did not interpret or prompt any questionnaire items to minimize the impact of technological barriers on the response process and ensure the independence of answers.

After collecting the questionnaires, the researchers systematically screened the data to ensure the validity of the final sample. First, respondents were verified for eligibility based on the preliminary screening information of the questionnaire regarding age range and experience with generative AI health consultations. Second, low-quality questionnaires were identified by evaluating the overall completion time, performance on attention-check items, and scale response patterns. Additionally, samples exhibiting obvious patterned responses, a high proportion of consecutive identical answers, or significant logical inconsistencies among key items were reviewed. Based on a comprehensive assessment, questionnaires that failed to meet these requirements or displayed clear indicators of low-quality responses were excluded, and valid samples were retained for subsequent analyses. In total, 1,060 questionnaires were distributed in this study, and 860 valid questionnaires were obtained and used as the basis for subsequent analysis. Table 2 presents the basic information of the respondents. The sample structure displays distinctly youthful, urbanized, and digital characteristics: respondents are primarily young members of the emerging older adults generation, most with stable employment histories and urban living backgrounds, and a generally stable social environment. This group typically possesses strong skills in operating smartphones and computers, providing the necessary technical foundation for accessing and using generative AI in health consultations. In terms of health status, most respondents rated themselves as average or good, while a certain proportion of older adults rated themselves unfavorably, indicating diverse health management needs and forming an intrinsic motivation for utilizing generative AI for health consultations. Correspondingly, at the behavioral level, most respondents have developed certain usage frequencies and interaction habits, which are mainly characterized by moderate and regular use. This suggests that respondents are not merely engaging in occasional experimentation with generative AI but have, to some extent, integrated it into their routine health information acquisition and management practices, demonstrating a strong alignment with the generative AI health consultation context on which this study focuses.

**Table 2:** Basic information statistics of survey participants.

Statistical item	Option	Frequency
Age	61–65 years old	511
	66–70 years old	264
	71–76 years old	85
Occupation	Government agency/public institution	190
	Enterprise	634
	Freelancer/self-employed individual	36
Residence	Tier-1 cities	234
	New tier-1cities	216
	Tier-2 cities	299
	Tier-3 and lower-tier cities	111
Living situation	Living alone	96
	Living with a spouse	545
	Living with children and other family members	219
Proficiency with mobile phones and computers	Highly proficient	254
	Fairly proficient	401
	Basic proficient	205
Health status	Relatively healthy	193
	Average	419
	Poor health	248
Frequency of weekly consultations	1–2 times	321
	3–5 times	496
	Almost every day	43
Duration of each consultation	Under 30 min	311
	30–60 min	419
	Over 60 min	130

## 5 Empirical result and discussion

### 5.1 Preliminary inspection

#### 5.1.1 Reliability and validity analysis

First, the reliability of the data was assessed. Table 3 shows that the overall Cronbach's alpha reached 0.865, meeting the standard. The test results for each dimension also fell within a reasonable range. This indicates that there is good internal consistency among the measurement items, the data collected are highly stable, and can support subsequent statistical analysis and model estimation.

**Table 3:** Reliability test.

Variable	Number of measurement items	Cronbach's alpha	Reliability
HC	3	0.856	High
IA	3	0.807	High
IP	3	0.852	High
ID	3	0.872	High
IT	3	0.849	High
IR	3	0.832	High
BA	3	0.859	High
PA	3	0.836	High
HA	3	0.863	High
HD	3	0.797	High
Totality	30	0.865	High

HC is generative AI health consultation, IA is attention to AI-generated health information, IP is perception of AI-generated health information, ID is discernment of AI-generated health information, IT is trust in AI-generated health information, IR is reliance on AI-generated health information, BA is intelligent boundary awareness, PA is algorithmic pandering awareness, HA is health autonomy orientation, HD is health decision quality, II is intelligent information.

The KMO test and Bartlett's test of sphericity were applied to assess the data suitability. According to Table 4, the KMO statistic is 0.885, indicating that the overall performance of the sample data is relatively good, and the result of Bartlett's test of sphericity also reached statistical significance. Overall, the validity of the data met the requirements for analysis and provided effective support for subsequent research.

**Table 4:** Validity test.

Test		Result
Kaiser-Meyer-Olkin measurement of sampling adequacy		0.885
Bartlett test of sphericity	Approximate chi-square	13214.049
	Degrees of freedom	435
	Statistical significance	0.000

#### 5.1.2 Convergent and discriminant validity

As shown in Table 5, the CR values of all constructs exceeded 0.70, indicating satisfactory composite reliability. Meanwhile, the AVE values of all constructs are above 0.50, suggesting that the latent constructs explain a substantial proportion of the variance in their respective measurement items and thus demonstrate good convergent validity. Overall, the measurement model meets the acceptable criteria for composite reliability and convergent validity, providing a sound measurement basis for the subsequent examination of structural relationships.

**Table 5:** Convergent validity.

Variable	CR	AVE
HC	0.845	0.646
IA	0.813	0.593
IP	0.854	0.661
ID	0.874	0.700
IT	0.854	0.661
IR	0.832	0.623
BA	0.829	0.619
PA	0.869	0.689
HA	0.820	0.603
HD	0.789	0.556

Discriminant validity was further assessed for the main study variables. As shown in Table 6, the square root of the AVE for each construct is greater than its correlation with the other constructs, thereby satisfying the criterion for discriminant validity. These results indicate that the constructs can be effectively distinguished from one another statistically, and that the measurement model demonstrates satisfactory discriminant validity.

**Table 6:** Discriminant validity.

Variable	HC	IA	IP	ID	IT	IR	BA	PA	HA	HD
HC	0.803									
IA	0.342	0.770								
IP	0.241	0.375	0.813							
ID	0.315	0.282	0.280	0.836						
IT	0.300	0.365	0.327	0.282	0.813					
IR	0.323	0.385	0.334	0.322	0.338	0.789				
BA	0.276	0.362	0.334	0.193	0.356	0.306	0.787			
PA	0.314	0.370	0.310	0.120	0.345	0.376	0.178	0.830		
HA	0.301	0.329	0.331	0.082	0.287	0.350	0.152	0.228	0.776	
HD	0.324	0.431	0.341	0.271	0.371	0.396	0.332	0.379	0.366	0.745

#### 5.1.3 Goodness-of-fit test

To assess the overall model fit with the sample data, the model fit was evaluated using three categories of indices: absolute fit, incremental fit, and parsimonious fit indices. The results are presented in Table 7. All fit indices met the commonly accepted reference criteria, indicating that the model effectively captured the relationships among the variables. Considering model complexity, the parsimonious fit indices remained within a reasonable range, suggesting that the model specification was concise and free of parameter redundancy. Overall, the hypothesized model demonstrated a good fit, with strong agreement between the theoretical framework and empirical data, providing a solid basis for subsequent path analysis and hypothesis testing.

**Table 7:** Fit index.

Metrics	Indicator	Adaptation criteria	Value	Conclusion
Absolute fit metrics	CMIN/DF	1 < CMIN/DF < 3	2.562	Ideal
	RMR	RMR < 0.05	0.031	Ideal
	GFI	GFI > 0.90	0.922	Ideal
	AGFI	AGFI > 0.90	0.908	Ideal
	RMSEA	RMSEA < 0.08	0.055	Ideal
Incremental fit metrics	NFI	NFI > 0.90	0.929	Ideal
	IFI	IFI > 0.90	0.948	Ideal
	TLI	TLI > 0.90	0.938	Ideal
	CFI	CFI > 0.90	0.947	Ideal
Parsimonious fit metrics	PGFI	PGFI > 0.50	0.710	Ideal
	PNFI	PNFI > 0.50	0.787	Ideal
	PCFI	PCFI > 0.50	0.803	Ideal

### 5.2 Model evaluation

#### 5.2.1 Main influencing path analysis

The test results (Table 8) show that generative AI health consultations have significant positive effects on multiple dimensions of smart information, which in turn further influence the health decision quality of the new generation of older adults. Moreover, the direction and significance of the main model pathways were largely consistent with the research hypotheses, indicating that generative AI health consultations do not simply increase sources of health information but deeply intervene in older users' attention to, understanding of, judgment of, and utilization of information, thereby impacting their health decision-making. This result reveals that the role of generative AI in health consultations is largely reflected in information processing. As consultation interactions deepen, the new generation of older adults will increasingly focus on information relevant to their health needs, gradually forming perceptions and understanding of the content. Simultaneously, when faced with AI-generated advice, they evaluate it by combining their own experience, existing knowledge, and other information sources, thereby forming different levels of discernment, trust, and reliance. Thus, smart information plays a crucial role in the generative AI health consultations and the quality of health decision-making. Whether generative AI can truly improve health decisions depends not only on its ability to provide information but also on whether that information can be effectively attended to, understood, evaluated, and adopted by users.

**Table 8:** Model path analysis and hypothesis verification.

Path relationship	Standardized path coefficient	Unstandardized path coefficient	Correlation	*P*	Conclusion
Attention to AI-generated health information < – generative AI health consultation	0.466	0.408	Positive	^***^	Assumption established
Perception of AI-generated health information < – generative AI health consultation	0.357	0.341	Positive	^***^	Assumption established
Discernment of AI-generated health information < – generative AI health consultation	0.405	0.483	Positive	^***^	Assumption established
Trust in AI-generated health information < – generative AI health consultation	0.413	0.392	Positive	^***^	Assumption established
Reliance on AI-generated health information < – generative AI health consultation	0.451	0.402	Positive	^***^	Assumption established
Health decision quality < – generative AI health consultation	0.084	0.074	Positive	^***^	Assumption established
Health decision quality < – attention to AI-generated health information	0.256	0.257	Positive	^***^	Assumption established
Health decision quality < – perception of AI-generated health information	0.135	0.124	Positive	^***^	Assumption established
Health decision quality < – discernment of AI-generated health information	0.098	0.097	Positive	^***^	Assumption established
Health decision quality < – trust in AI-generated health information	0.187	0.173	Positive	^***^	Assumption established
Health decision quality < – reliance on AI-generated health information	0.225	0.222	Positive	^***^	Assumption established

*P* represents significance, and ^***^ indicates significance at the 0.001 level.

#### 5.2.2 Mediating effect analysis

The Bootstrapping method was used to examine the mediating roles of intelligent information attention, perception, discernment, trust, and reliance in the relationship between generative AI health consultation and health decision quality. The results (Table 9) show that the confidence intervals for all five mediation paths do not include 0, indicating that these dimensions play significant mediating roles, thereby supporting the relevant hypotheses. This demonstrates that the impact of generative AI health consultation on the quality of health decisions does not simply stem from the supply of information itself but rather requires users to engage in ongoing exposure, cognitive processing, and judgment of intelligent information. Simultaneously, the establishment of these mediating effects further reveals the key mechanism by which generative AI health consultations translate technology use into decision-making influence. Although generative AI can provide health information in an immediate, interactive, and personalized manner, such information only truly participates in health decision-making when integrated into the information processing and judgment systems of the new generation of older adults. Thus, intelligent information becomes an important link connecting technological capability and decision outcomes, allowing AI-generated content to gradually transform from external information output into resources that are understandable, evaluable, and actionable for decision-making. This process also demonstrates that the new generation of older adults are not passive recipients of technological advice but actively construct health decisions through their own judgment and use. Therefore, the key for generative AI to improve the quality of health decisions lies not only in whether it can generate information, but more importantly, in whether the information can be actively processed and transformed into effective decision-making grounds.

**Table 9:** Mediation effect test.

Relationships	Point estimate	Product of coefficients		Bootstrapping					
				Bias-Corrected 95% CI			Percentile 95% CI		
		S.E.	*Z*	Lower	Upper	*P*	Lower	Upper	*P*
Indirect effects									
HC → IA → HD	0.105	0.024	4.375	0.066	0.161	0.001	0.065	0.158	0.001
HC → IP → HD	0.042	0.014	3.000	0.021	0.075	0.001	0.019	0.072	0.001
HC → ID → HD	0.047	0.016	2.938	0.020	0.070	0.001	0.017	0.069	0.001
HC → IT → HD	0.068	0.017	4.000	0.040	0.106	0.001	0.039	0.105	0.001
HC → IR → HD	0.089	0.020	4.450	0.058	0.139	0.000	0.054	0.132	0.001
Total IE	0.351	0.050	7.020	0.230	0.426	0.001	0.228	0.425	0.001

#### 5.2.3 Moderating effect analysis

This study aims to reveal the boundary conditions of health decision quality among the new generation of older adults and to further test the moderating roles of intelligent boundary awareness, algorithmic pandering awareness, and health autonomy orientation in the relationship between intelligent information and health decision quality. As shown in Table 10, all three variables significantly and positively moderated the impact of intelligent information on health decision quality, indicating that whether generative AI health information can be transformed into high-quality decisions depends not only on the content of the information but also on the new generation of older adults' cognition of technological boundaries, algorithmic logic, and decision-making roles. Specifically, intelligent boundary awareness helps the new generation of older adults recognize the scope and limitations of AI in health consultations, preventing them from treating intelligent information as absolute and enabling them to maintain judgment boundaries in use. Algorithmic pandering awareness changes users' interpretation of AI-generated content; when the new generation of older adults realizes that algorithms may accommodate their preferences and interaction histories, they evaluate the objectivity and applicability of information more cautiously rather than merely accepting answers that match their expectations. Health autonomy orientation reflects the subjectivity of the new generation of older adults in decision-making; those with stronger autonomy tend to weigh AI recommendations in light of their own needs, experiences, and context, treating them as auxiliary references rather than substitutes for personal judgment. Thus, the effect of intelligent information on the quality of health decisions is jointly moderated by individual technological cognition and health autonomy. The three moderating variables together reveal important boundaries for the decision-making value of generative AI health consultations: high-quality intelligent health decisions are not based on simply accepting AI information but rather emerge from a dynamic balance between technology utilization and individual autonomous judgment.

**Table 10:** Moderation effect test.

Path relationship	Standardized path coefficient	Unstandardized path coefficient	C.R.	Correlation	*P*
HD < – II × BA	0.200	0.150	3.788	Positive	^***^
HD < – II × PA	0.207	0.155	4.236	Positive	^***^
HD < – II × HA	0.190	0.144	3.520	Positive	^***^

*P* represents significance, and ^***^ indicates significance at the 0.001 level.

### 5.3 Fuzzy-set qualitative comparative analysis

#### 5.3.1 Variable selection and data calibration

Taking intelligent information attention, perception, discernment, trust, reliance, intelligent boundary awareness, algorithmic pandering awareness, and healthy autonomous orientation as condition variables, and health decision quality as the outcome variable, a configurational model was constructed to analyze the interaction among different conditions and to identify multiple pathways and combinatorial mechanisms that influence health decision quality. Before the configurational analysis, direct calibration was used to perform fuzzy set calibration of the raw data. Based on the sample distribution, following the common practice, 95% was selected as the full-membership anchor point, 50% as the crossover anchor point, and 5% as the full non-membership anchor point. thereby converting each variable into fuzzy set membership scores ranging from 0 to 1. The calibration results are presented in Table 11. This process not only preserves the relative differences among samples but also clarifies each case's membership status in different condition sets, providing a standardized data basis for subsequent necessary condition testing and sufficient condition configuration analysis.

**Table 11:** Variable calibration.

Variable	Complete membership point	Intersection point	Complete non-membership point
IA	6.0	5.4	1.9
IP	6.2	5.3	2.0
ID	5.7	2.5	1.5
IT	6.2	5.3	2.0
IR	6.2	5.3	2.0
BA	5.8	2.3	1.5
PA	5.7	2.4	1.6
HA	5.9	2.5	1.6
HD	6.2	5.4	1.9

#### 5.3.2 Necessary condition analysis and truth table construction

Prior to conducting the configurational analysis of conditions, each condition was examined to determine whether it constituted a necessary condition for health decision quality. According to the general criterion for necessity analysis, a consistency level of no < 0.90 can be regarded as a necessary prerequisite for the outcome. Table 12 shows that the consistency of all individual conditions and their negated states is below 0.90, failing to reach the threshold for determining necessity. This indicates that health decision quality is not determined by any single condition alone; the presence or absence of any one condition does not constitute a necessary prerequisite for an outcome. These results reveal that the formation of health decision quality exhibits clear features of multiple conjunctural causation, necessitating further exploration from the perspective of condition combinations to examine synergistic effects and realization paths among different factors.

**Table 12:** Necessity test of antecedent condition variables.

Predictor variable	Consistency	Coverage
IA	0.796	0.776
~IA	0.440	0.677
IP	0.762	0.773
~IP	0.490	0.711
ID	0.620	0.705
~ID	0.635	0.797
IT	0.779	0.783
~IT	0.478	0.701
IR	0.749	0.794
~IR	0.518	0.708
BA	0.643	0.714
~BA	0.642	0.829
PA	0.643	0.717
~PA	0.646	0.829
HA	0.620	0.704
~HA	0.649	0.816

Based on necessary condition testing, truth table analysis was further employed to examine the relationship between combinations of conditions and health decision quality to identify sufficient configurations leading to high decision quality. The truth table summarizes the distribution of cases across different combinations of conditions and their corresponding outcomes, revealing the linkage patterns between various configurations and outcomes. To reduce the interference of low-frequency combinations with the research conclusions, the case frequency threshold was set at 10, and the consistency and PRI thresholds were set at 0.85 and 0.75, respectively, to ensure that the screening results were representative and reliable. After processing according to these criteria, a total of 17 condition configurations with actual case support were obtained (Table 13).

**Table 13:** Truth table summary.

IA	IP	ID	IT	IR	BA	PA	HA	Number	HD
1	1	0	0	1	1	1	1	13	1
0	1	1	1	1	1	1	1	11	1
0	1	1	1	1	1	0	0	12	1
0	1	0	1	1	1	0	0	12	1
0	1	0	1	1	1	1	1	17	1
0	1	0	1	1	1	1	0	18	1
1	1	1	1	1	1	1	1	20	1
0	1	0	1	1	1	0	1	12	1
0	1	0	1	1	0	1	0	11	1
1	1	1	1	1	1	1	0	10	1
1	1	0	1	1	1	1	1	23	1
1	1	1	1	1	1	0	1	20	1
1	1	1	1	1	1	0	0	17	1
1	1	1	1	1	0	0	1	11	1
1	1	0	1	1	1	0	1	10	1
1	1	0	1	1	1	1	0	15	1
1	1	0	1	1	1	0	0	13	1

#### 5.3.3 Configuration analysis

There are seven pathways for health decision-making among the new generation of older adults, from which three typical patterns have been distilled (Table 14).

**Table 14:** Configuration classification.

Prerequisite	Mode 1			Mode 2		Mode 3	
	Configuration 1a	Configuration 1b	Configuration 1c	Configuration 2a	Configuration 2b	Configuration 3a	Configuration 3b
IA			⊗		•	•	•
IP	●	●	●	●	●	●	●
ID	⊗		⊗		⊗	⊗	•
IT	•	•	•	•		•	•
IR	•	•	•	•	•	⊗	⊗
BA	•	•		•	•		
PA		⊗	●	●	●	●	⊗
HA		⊗	⊗	•	•	⊗	●
Consistency	0.891	0.913	0.920	0.925	0.932	0.920	0.915
Raw coverage	0.323	0.286	0.190	0.288	0.251	0.190	0.264
Unique coverage	0.002	0.003	0.015	0.001	0.024	0.015	0.026
Solution consistency	0.883						
Solution coverage	0.762						

● Core condition, • Supplementary condition, ⊗ Lack of core condition, ⊗ Lack of auxiliary condition.

Mode 1 is the trust-reinforced type of relationship. This mode includes three typical configurations: configuration 1a is IP^*^~ID^*^IT^*^IR^*^BA, configuration 1b is IP^*^IT^*^IR^*^BA^*^~PA^*^~HA, and configuration 1c is ~IA^*^IP^*^~ID^*^IT^*^IR^*^PA^*^~HA. Among these, intelligent information perception, trust, and reliance are the key factors driving health decisions among the new generation of older adults. The core feature is that, through ongoing generative AI health consultations, the new generation of older adults establishes a stable relationship of information recognition and trust, allowing intelligent information to be continuously integrated into their health judgments and decision-making. Further comparison of different configurations shows that even if there are differences among individuals in proactive engagement, information discernment, or technical boundary cognition, strong information perception, trust, and reliance can still complement other conditions to collectively achieve high decision quality, demonstrating evident characteristics of condition substitution and multipath equivalence. This mode represents a decision-support mechanism driven by stable trust. When the new generation of older adults continuously perceives the value of generative AI health information and forms strong trust and a stable usage tendency, AI shifts from a general information acquisition tool to a critical reference in daily health decision-making. This mechanism is especially applicable to scenarios with strong continuity and repetitive information needs, such as daily health management, lifestyle adjustments, and auxiliary judgment of long-term health issues. Repeated human-AI interaction helps consolidate this usage relationship, allowing intelligent information to remain consistently involved in decision-making, thus exerting a cumulative supportive effect.

Mode Two is the autonomous adaptive type. This mode contains two typical configurations: configuration 2a is IP^*^IT^*^IR^*^BA^*^PA^*^HA, and configuration 2b is IA^*^IP^*^~ID^*^IR^*^BA^*^PA^*^HA. Among these, intelligent information perception, intelligent information reliance, intelligent boundary awareness, algorithmic pandering awareness, and health autonomy orientation are the key conditions influencing the health decision-making of the new generation of older adults. Its core characteristic lies in the fact that, while continuously utilizing generative AI health information, the new generation of older adults can maintain a basic understanding of the technological capability boundaries and operational characteristics of algorithms, as well as preserve their subjectivity in health decision-making. Therefore, dependence in this mode is not a passive acceptance of intelligent information but rather a technology-use relationship characterized by boundary awareness and self-regulation. This mode is more applicable to health decision-making scenarios with higher information complexity, requiring multiple trade-offs, or where AI recommendations have a certain degree of uncertainty. In such cases, generative AI primarily plays an auxiliary role in analysis and information support rather than directly replacing individual decision-making. Thus, this mode represents a human-AI collaborative decision-making approach that is dependent but not blindly, making use of technology yet bounded, with the key lying in the dynamic balance between the use of intelligent technology and health autonomy.

Mode Three is the cognitive adoption type. This mode includes two typical configurations: configuration 3a is IA^*^IP^*^~ID^*^IT^*^~IR^*^PA^*^~HA, and configuration 3b is IA^*^IP^*^ID^*^IT^*^~IR^*^~PA^*^HA. Among these, intelligent information attention, perception, and trust are the key conditions influencing the health decisions of the new generation of older adults. Its core feature is that the new generation of older adults actively pay attention to and understand health information provided by generative AI, and after establishing basic trust, incorporate valuable content into their health judgments without the need to establish continuous reliance on technology. This mode does not depend on a long-term and stable human-AI usage relationship but instead emphasizes the recognition and adoption of information value by the new generation of older adults, reflecting a decision-making approach characterized by cognitive acceptance as a prerequisite and selective adoption as its hallmark. Therefore, it is more applicable to highly targeted, episodic, or situational health consultation scenarios, such as health indicator interpretation, consultation of specific health problems, and lifestyle choices. In such contexts, generative AI mainly serves as a source of cognitive support and decision-making reference; its value lies in expanding information and supporting judgment rather than being a continuously relied-upon decision-making agent. Consequently, this mode reveals the pathway through which generative AI health information is attended to, understood, recognized, and selectively incorporated into decision-making.

In summary, the three modes reveal multiple equivalent pathways by which the new generation of older adults can achieve high-quality health decisions with the aid of generative AI. The quality of health decision-making is not determined by any single factor but is the result of the interplay among intelligent information characteristics, technological cognition, and individual autonomy, with clear relationships of complementarity and substitution among different conditions. This means that there is no single optimal way to use generative AI health consultations; the new generation of older adults can make effective decisions through different information processing routes and human-AI interaction patterns. More importantly, all three models jointly demonstrate that generative AI is shifting from a mere information tool to a collaborative participant in health decision-making. However, this collaboration does not involve technology replacing human judgment but rather emerges from the adaptation and balance between technological capabilities and individual cognition and autonomy. Therefore, the key to enhancing the decision-making value of generative AI health consultations lies in building mechanisms for human-AI collaboration that are tailored to users' cognitive characteristics and decision-making styles, rather than simply reinforcing the use of technology. This further indicates that smart health decision-making among the new generation of older adults is markedly heterogeneous and context-dependent, providing a more systematic configurational perspective for understanding how generative AI can be effectively transformed into health decision-making competence in older adults.

## 6 Conclusion and implication

This study focuses on the emerging phenomenon of the new generation of older adults using generative AI for health consultation and constructs a theoretical model of how generative AI intervenes in health information processing and influences the quality of health decision-making. It systematically examines the mechanisms of action, boundary conditions, and multiple implementation pathways. The main findings are as follows: (1) Generative AI health consultation influences decision-making not only by increasing information supply but also by reshaping how the new generation of older adults processes and utilizes intelligent information, thereby improving the quality of health decisions. This indicates that the health value of generative AI has extended from an information acquisition tool to an integral part of the individual decision-making process. (2) Intelligent boundary awareness, algorithmic pandering awareness, and health autonomy orientation significantly moderate the transformation of intelligent information into health decision quality, demonstrating that the decision-making effectiveness of generative AI has clear cognitive and agentic boundaries. The formation of high-quality intelligent health decisions inherently relies on effective coordination among technology utilization, rational cognition, and autonomous judgment. (3) This study identified three high-quality health decision-making patterns: trust-reinforced, autonomous adaptive, and cognitive adoption, revealing the multipath equivalence in how generative AI empowers health decisions. This finding challenges the linear logic of single-factor explanations for intelligent health decisions, indicating that the decision-making effectiveness of generative AI depends on differentiated adaptation among information cognition, technology relationships, and individual autonomy. The core value of generative AI in older adult health contexts does not lie in replacing individual judgment but in forming a decision support relationship adapted to users' cognitive characteristics and capacity for autonomy. Therefore, in constructing generative AI health services for the new generation of older adults, the key lies in helping them develop the ability to utilize AI effectively without being constrained by it, thus advancing intelligent health services from technological accessibility to decision-making empowerment.

In response to the growing normalization of generative AI health consultations among the new generation of older adults, intelligent health services should shift from simply providing tools to empowering users. First, enhancing health decision-making ability should be the core goal of health education. This can be achieved through ongoing digital health education and contextualized guidance to strengthen the capacity of the new generation of older adults to think independently, integrate information, and make rational judgments using intelligent technologies, thereby ensuring that technology truly serves autonomous individual decision-making. Second, generative AI platforms should assume greater technical responsibility by increasing information transparency, interpretability, and risk warning capabilities, thereby reducing potential interference with health judgment caused by algorithmic catering, information bias, and overreliance. Finally, families, communities, professional health service institutions, and intelligent platforms should be encouraged to form a multistakeholder collaborative support system, providing flexible, tiered, and sustainable decision support for new-generation older adults with diverse cognitive profiles and usage habits. Accordingly, the development focus of generative AI health services should move beyond merely enabling the new generation of older adults to use technology, shifting further toward empowering them to make better judgments with technology, and ultimately establishing an intelligent health decision-making ecosystem that centers on individual autonomy, is underpinned by technological support, and safeguarded by multi-party collaboration.

However, this study has certain limitations. The sample mainly focuses on the new generation of older adults with experience in generative AI health consultations. Given the specific characteristics of the participants, the findings are primarily applicable to relatively younger, digitally engaged older adults who have experience using generative AI for health-related information and decision making. Caution should therefore be exercised when extending the findings to the broader older population, particularly those with limited digital literacy or no prior experience with generative AI. Future research could include older adults with more diverse levels of digital experience and technology use and further examine the ongoing evolution of generative AI health consultation behaviors and address differences in health decision-making mechanisms across diverse population characteristics, usage contexts, and stages of technological development, thereby enhancing the generalizability and explanatory power of the research findings.

## Data Availability

The raw data supporting the conclusions of this article will be made available by the authors, without undue reservation.
